# Landmark Prediction of Survival for Breast Cancer Patients: A Case Study in Tehran, Iran

**Published:** 2019-12

**Authors:** Behnaz ALAFCHI, Leili TAPAK, Omid HAMIDI, Jalal POOROLAJAL, Hossein MAHJUB

**Affiliations:** 1. Department of Biostatistics, School of Public Health, Hamadan University of Medical Sciences, Hamadan, Iran; 2. Modeling of Noncommunicable Diseases Research Center, School of Health, Hamadan University of Medical Sciences, Hamadan, Iran; 3. Department of Science, Hamedan University of Technology, Hamedan, Iran; 4. Research Center for Health Sciences, School of Public Health, Hamadan University of Medical Sciences, Hamadan, Iran; 5. Department of Epidemiology, School of Public Health, Hamadan University of Medical Sciences, Hamadan, Iran

**Keywords:** Breast neoplasms, Survival analysis, Landmarking, Dynamic prediction, Cohort studies

## Abstract

**Background:**

Breast cancer is the first non-cutaneous malignancy in women and the second cause of death due to cancer all over the world. There are situations where researchers are interested in dynamic prediction of survival of patients where traditional models might fail to achieve this goal. We aimed to use a dynamic prediction model in analyzing survival of breast cancer patients.

**Methods:**

We used a data set originates from a retrospective cohort (registry-based) study conducted in 2014 in Tehran, Iran, information of 550 patients were available analyzed. A method of landmarking was utilized for dynamic prediction of survival of the patients. The criteria of time-dependent area under the curve and prediction error curve were used to evaluate the performance of the model.

**Results:**

An index of risk score (prognostic index) was calculated according to the available covariates based on Cox proportional hazards. Therefore, hazard of dying for a high-risk patient with breast cancer within the next five years was 2.69 to 3.04 times of that for a low-risk patient. The value of the dynamic C-index was 0.89 using prognostic index as covariate.

**Conclusion:**

Generally, the landmark model showed promising performance in predicting survival or probability of dying for breast cancer patients in this study in a predefined window. Therefore, this model can be used in other studies as a useful model for investigating the survival of breast cancer patients.

## Introduction

Breast cancer is the first non-cutaneous malignancy in women and the second cause of death due to cancer all over the world (1–4). Breast cancer accounts for more than 40% of all new case cancers among women by age 40 (4). Around 7% of breast cancers are diagnosed in women in this age group, of which 2.4% are in women less than 35, and 0.65% are in women less than 30. Survival rates of this group are worse compared to those in older women (5). By age 40 yr, the average risk for developing this cancer is 1/173 (6, 7). Disease-free survival of cancer patients that can be defined as the time duration between the onset of disease and occurrence of metastasis/death, is the most common criterion to evaluate their treatment. There have been identified different factors affecting disease-free survival (8, 9). Surgery and chemotherapy in younger women and hormonal treatments such as tamoxifen in postmenopausal women with early breast cancer can improve disease-free survival of the patients (10–13). Moreover, survival of the patients who are “human epidermal growth factor receptor 2 (HER2)”, “estrogen receptor (ER)” positive and “progesterone receptor (PR)” positive and receive complementary therapies were higher than those who do not receive these therapies (14).

Understanding the prognostic factors influencing survival is of great importance in treatment and care process of the patients who developed breast cancer. Although several prognostic factors affecting survival of the breast cancer patients have been identified by different studies (1, 4, 9, 15), there are inconsistencies among them. On the other hand, the dynamic effects of these covariates on survival of breast cancer patients have not been investigated by these studies. In addition, patients' characteristics across counties might be different resulting in different factors.

There are situations where researchers are interested in dynamic prediction of survival of patients (i.e. in calculating the predictive distribution in predefined time points given the history of covariate(s)/event(s) until that moment) (16). For example, one of the main objectives in survival analysis may be to determine the effect of response to a treatment like surgery or chemo-therapy on survival of the patients usually done by comparing survival of say two groups of responders and non-responders. However, only those responders that survive until time of response have the potential of belonging to the responder group. These individuals are immortal for some time (immortal time bias) that gives them an unfair survival advantage. However, this might not be known at baseline and in survival analysis, making groups based on an event that happens in the future is not allowed. There are two alternative approaches of time-dependent covariates and landmark (that considers response at fixed time point and removes patients with censor or event before this landmark point from analysis). Landmarking is a useful approach that can be applied in this situation as it can dynamically adjust the predictive survival model for the data over the follow-up period. In this dynamic prediction process, updating is accomplished through fitting models for those subjects that are still at risk at the landmark point directly (17, 18). The method calculates the aforementioned probabilities based on the data by choosing those individuals who are at risk at that special instant and utilized only the available data at that special moment to make predictions (16, 19, 20). The landmark methodology has interesting properties as it does not require an intricate structure of modeling, while it provides easy prediction rules (17).

In this study we used landmark model to obtain dynamic prediction of survival of breast cancer patients.

## Methods

### Ethical Approval

This study was approved by the Research Ethics Committee of Hamadan University (No. IR.UMSHA.REC.1398.568). Written informed consent was obtained from all participants.

### Data source

We used a data set originates from a retrospective cohort (registry-based) study that was conducted in 2014 in Tehran. We used the information of patients who developed breast cancer (International Classification of Diseases for Oncology 3^rd^ edition sites C50.0–C50.9) and registered with the Comprehensive Cancer Control Center associated with Shahid Beheshti University of Medical Sciences, Tehran, Iran from 1998 to 2013. All patients diagnosed pathologically and the patients with unknown pathology were excluded from analysis.

We selected 9 risk factors that are believed associated with survival of breast cancer patients, including age, grade (1 to 3), stage (I to IV), metastasis (yes/no), Estrogen receptor (ER as negative or positive), Progesterone receptor (PR as negative or positive), Human epidermal growth factor receptor 2 (HER2 as negative or positive), Pathological type (Ductal/lobular carcinoma in situ, Invasive lobular carcinoma and Invasive ductal carcinoma), and Surgical approach (Modified Radical Mastectomy and Breast-conserving surgery).

### Landmarking

We consider the covariates vector as *X* that their effects may change over time. The main propose is to obtain dynamic prediction of survival up to a certain horizon (*t* *_hor_*), *S*(*t_hor_*|*X*), that is of main interest clinically and we will use the landmark model to achieve this.

The basic landmark model is defined as

$$\begin{array}{cc} h(t|X\;,t_{LM})=h_{0}(t|t_{LM})\text{exp}(X\;\beta_{LM}) & t_{LM}\leq t\leq t_{hor} \end{array}$$

where *t_LM_* stands for the landmark point. We will select a set of prediction time points {*s*_1_, …,*s_L_*} as the landmark points, e.g. we select an interval [*s*_1_, *s_L_*] and take equidistant grade of points on this interval. Let β_LM_ (s) shows a weighted average of *β*(*t*) over [*s*, *t_hor_*] that varies with s smoothly. Two different forms of pseudo-likelihoods exist that can be used to fit models and to estimate *β_LM_* (*S*) . Both of them are inspired by creating new data sets. The first approach uses the super data set that described as follows:

Step 1: To define a window of width *w* that we want to consider the probability of failure within that window. The choice of the *w* depends on the length of follow up, the overall prognostics, and the purpose of study,
Step 2: To select a set of prediction time points {*s*_1_,…, *s_L_*} as the landmark points,
Step 3: To generate a prediction data set for each landmark point, each of {*s*_1_,…, *s_L_*}, by truncation and administrative censoring. These prediction data set are constructed by selecting those individuals who are at risk at s,
Step 4: To merge all generated data sets in step 3 into a “super data set”.

In the super data set, each subset corresponding to s is considered as a stratum. Passing across strata corresponds to sliding the mentioned window over time. Then the landmark model is a simple Cox model with stratification on the landmark points,

$$h(t|X\;,s)=h_{0}(t|s)\text{exp}(X\beta_{LM}(s)),$$

with unspecified baseline hazard and a model (*e.g.* linear model) for *β_LM_* (*S*) (*β_LM_* (*S*)= *f* (*S*)*θ*, that *f* (*S*) is a set of basis function (contain constant, linear, and quadratic functions) and *θ* is vector of parameters).

Although fitting this model can describe well how *β* *_LM_*(*S*) varies over time, it is not practically useful because the baseline hazard should be estimated for each stratum, separately. This baseline hazard estimate by,

$${\overset{⌢}{h}}_{0}(t_{i}|s)=\frac{1}{\sum_{t_{j^{\geq t_{i}}}}\text{exp}(x_{j}{\overset{⌢}{\beta }}_{LM}(s))}.$$

This baseline hazard varies continuously with *s* through *f*(*S*) in *β_LM_*(*S*), *i.e.*, a set of continuous functions. In the second approach the dependence of *ĥ*_0_ (*t*_i_|*S*) on *s* is modeled directly as follows,

$$h(t|X\;,s)=h_{0}(t)\text{exp}(X\;\beta_{LM}(s)+\gamma (s)).$$

This model can be fitted by using an unstratified model that a landmark term is added to the model. In this approach delayed entry is considered. In fact, an individual that enters to the study at *s* will get a record for each *S* ≤*t_i_* (*t_i_* is the time of failure for this person). In the data set used in this approach, each individual that is at risk at *t_i_*, will be presented *n_is_*=#(*S*≤*t_i_*) times in the data set. Therefore, this data set will be much bigger than the super data in the first approach (16, 18). In this study we calculated prognostic index by using covariates and then use it as X in the model.

We used dynamic C-index computed via taking an average over event times in the window, Brier score and time-dependent area under the ROC curve (Auc (t)) were used as evaluation criteria of the used model.

## Results

The information of 550 patients with breast cancer was used in the present study. Table 1 illustrates the patients' characteristics. The mean (SD) age of patients at diagnosis was 47.86 (11.79) yr (with minimum and maximum of 17 and 84 yr respectively). The majority of patients was at stage II (41.60%), presented with grade II (52.36%) and did not experience metastasis (84.91%). Moreover, most of the patients were ER+ (71.27%), PR+ (68.36%), HER2- (76.36%), diagnosed with pathological type of invasive ductal carcinoma (90.19%) and underwent breast-conserving surgery (65.09%) (Table 1).

**Table 1:** Characteristics of the patients with breast cancer (n=550) and the adjusted effects of clinical risk factors on survival

***Variable***	***Number (%) or mean (sd)***	***HR***	**P*-value***
Stage			
I	110 (20.00)		
II	228 (41.46)	2.51	0.087
III	188 (34.18)	2.35	0.095
IV	24 (4.36)	9.04	<0.001
Grade			
1	66 (12.00)		
2	288 (52.36)	0.66	0.461
3	196 (35.64)	1.23	0.715
Metastasis			
No	467 (84.91)		
Yes	83 (15.09)	12.51	<0.001
Estrogen receptor			
Negative	158 (28.73)		
Positive	392 (71.27)	0.52	0.056
Progesterone receptor			
Negative	174 (31.67)		
Positive	376 (68.36)	1.18	0.630
Human epidermal growth factor receptor 2			
Negative	420 (76.36)		
Positive	130 (23.64)	1.37	0.183
Pathological type			
Ductal/lobular carcinoma in situ	29 (5.27)		
Invasive lobular carcinoma	25 (4.54)	0.68	0.760
Invasive ductal carcinoma	496 (90.19)	1.83	0.557
Surgical approach			
Modified Radical Mastectomy	192 (34.91)		
Breast-conserving surgery	358 (65.09)	1.35	0.260
Age	47.86 (11.79)	1.05	<0.001

HR: Hazard Ratio; SE: Standard Error

**Fig. 1:**
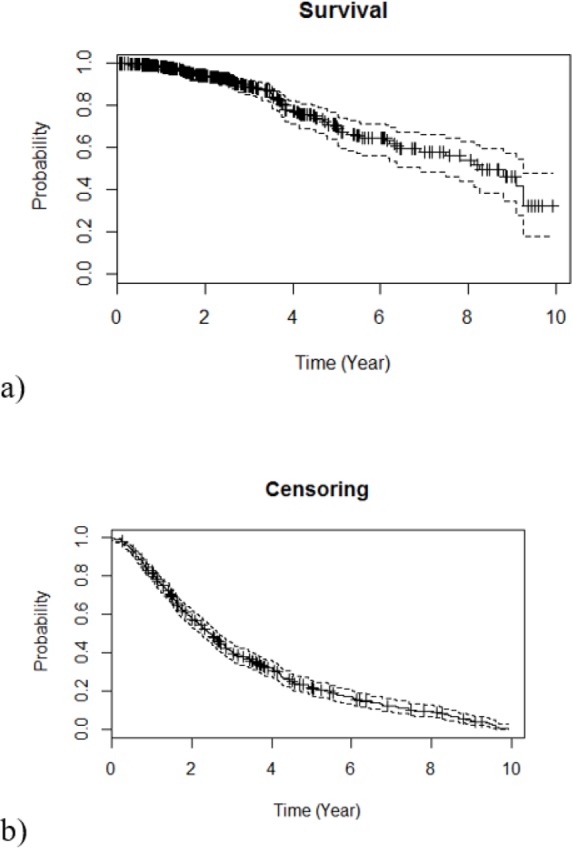
Survival and censoring functions for breast cancer data

Fig. 1 (a) and (b) shows the Kaplan-Meier estimates of both survival and censoring function plots. The probability of being alive for the patients was greater than 0.8 over the first four years and after this time it tends to diminish Fig. 1(a). The survival curve appears to be stabilized at a long term survival rate (after 9 years) of about 30%. The censoring curve shows that the median follow-up in the data set is less than 3 years. Moreover, as illustrated in Fig.1 (b), the probability of being censored after eight years tends towards zero.

We fitted Cox proportional hazards (PH) model with all predictors in the model. The adjusted effects of the used risk factors on survival in a Cox model are provided in Table 1. Stage, metastasis and age were of statistically significant. We computed prognostic index using the covariates for all individual (*PI*=(*X*–*X̄*)′*β̂*). The mean and standard deviation of PI was 0 and 1.44 respectively. PI showed a time-varying effect (*P*=0.020).

**Fig. 2:**
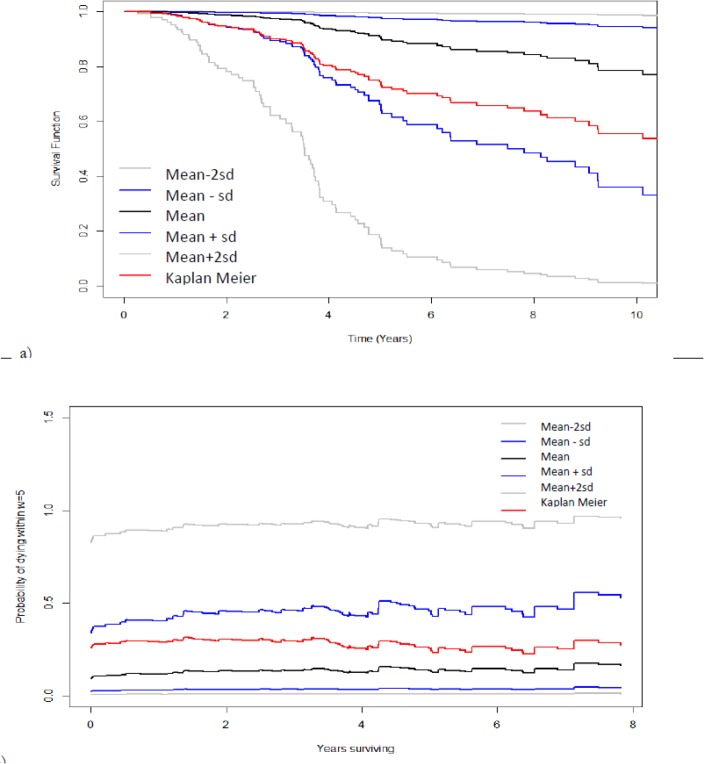
**a)** Predicted survival curves for different values of the prognostic index; **b)** five year dynamic probabilities of dying based on the proportional baselines landmark model, for the breast cancer data

Fig. 2 (a), shows the estimated survival curves (derived from the Cox model) for different range of the distribution of PI (*PĪ*±*sd*(*PI*), *PĪ*, and *PĪ*±2*sd*(*PI*)) . The estimated 10-year survival probabilities were 79% for mean PI (model-based estimate) and 53% for overall (Kaplan-Meier) survival. Fig. 2 (b) also illustrates the dynamic effect of the prognostic index which shows the probability of dying within a window of 5 years. The curves start to increase gradually after 4 years of follow-up. In the landmark analysis, a window of w=5 years was selected. In the Supermodel (refers to the stratified supermodel), landmarks points were selected between s_1_ =0 and s_L_ =3 (on an equidistant grid points with distance 0.1). The chosen time functions were f_1_(s) ≡ 1, f_2_(s) = s/3 and f_3_(s) = (s/3)^2^ to present constant, linear, and quadratic effects, respectively. The results of the regression coefficients of the landmark model were provided in Table 2 for breast cancer data with the prognostic score as predictor.

**Table 2:** Landmark model for the breast cancer data

***Part of model***	***Covariates***	***Time function***	***B***	***SE***
stratified				
*β_LM_*(*S*)	Prognostic index	Constant	1.031	2.803
		Linear	0.086	1.090
		quadratic	−0.097	0.908
Proportional hazards				
*β_LM_*(*S*)	Prognostic index	Constant	1.032	2.806
		Linear	0.079	1.083
		quadratic	−0.090	0.914
*γ*(*S*)		Linear	−0.175	0.839
		quadratic	0.195	1.216

**Fig. 3:**
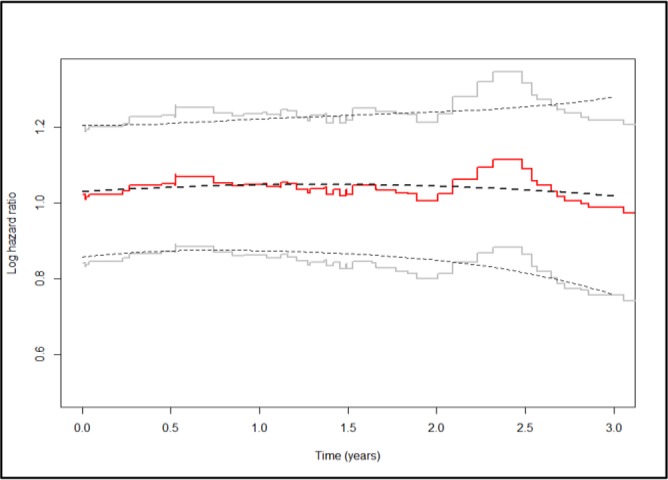
Landmark effects and pointwise 95% confidence intervals for the prognostic index of Table 2 in the breast cancer data for 5-years prediction window (note: The dashed lines stand for supermodel and the solid lines stand for the crude model)

Providing information about the regression effects, the stratified landmark can be applied for giving prediction at the selected landmark point. Dynamic prediction for the time points different from these landmark points is also possible for the supermodel through replacing the stratified baseline hazards with a single baseline hazard and considering additional effects via time functions in the form of g_j_(s). The results of the fitting the supermodel with g_1_(s) = s/3 and g_2_(s) = (s/3)^2^ were provided in Table 2 under PH model. Fig. 3 displays the estimated hazard ratio exp(*γ*(*S*)) as well as the baseline hazard (at s = 0) that reflects the landmark effects for the prognostic index of breast cancer patients (as well as their pointwise 95% confidence intervals). Crude refers to the results of stratified parts of Table 2 and it is related to the estimates obtained based on the separate landmark data sets at each of the time points. Supermodel stands for the stratified model with landmarks that were selected on equidistant grid points.

**Fig. 4:**
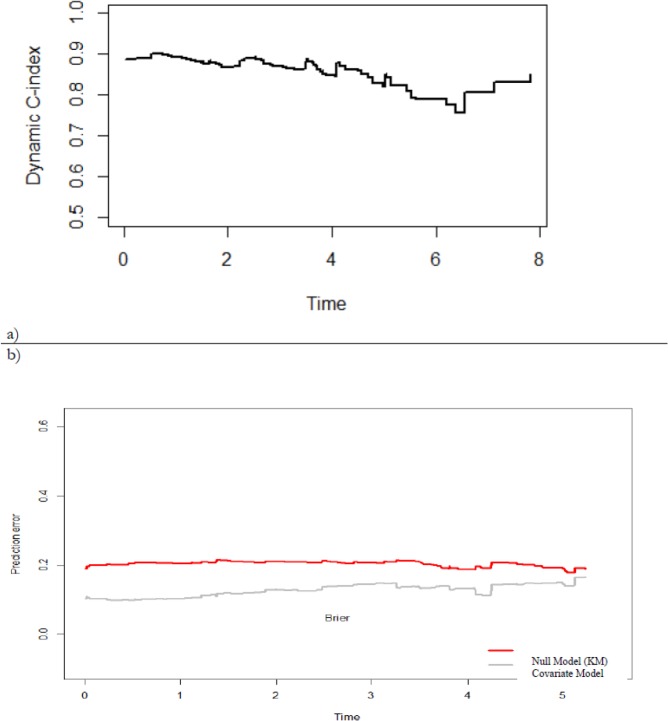
Evaluation criteria **(a)** Dynamic C- for 5-years prediction window; **(b)** Dynamic prediction error curve for Brier scores for 5-years prediction window

We assessed the accuracy and prediction power of the model using dynamic versions of C-index and Brier score. Fig. 4 (a) and (b) show the results for *w*=5. It is clear from curves that the landmark model prediction was adequate. The value of the dynamic C-index was greater than 0.75 overtime. For the prognostic index, we obtained C=0.89, indicating a promising predictive accuracy. Fig. 4 (b) shows the average prediction error overtime of the prognostic model of Table 2, as well as the mean prediction error for the Kaplan-Meier estimate (null model). The prediction error of the landmark model lies always under that of the KM one indicating a better prediction power. Nevertheless, the discriminative ability slowly decreases over time.

## Discussion

Fitting only a standard Cox model provides a rational prediction for survival of the patients up to special time, even without holding the PH assumption (21). Nevertheless, in reality, there are situations that dynamic prediction is of interest and using this model might be disastrously wrong and it may lead to misleading conclusions. We analyzed survival of breast cancer patients using landmarking (20) to provide a dynamic perspective towards survival of these patients. The emphasis of this method is to answer questions such as “Is the patient cured?” or “How long does a patient survive after treatment?” (18) that are valuable greatly in public health and survivallence systems.

To this end, prognostic index was calculated for the patients defined as the joint effects of all potential predictors in that model with time-invariant effects including age, stage and metastasis. Considering prognostic index had time varying effect on survival of the patients, the log hazard ratios were calculated using landmarking (changes over time) which was significantly different from zero for all landmark time points. The log hazard ratios fluctuate around 1 over time indicating that for those patients who are high risk according to the prognostic index values had greater hazards of dying within a window of 5-year compared with the low-risk patients. Therefore, hazard of dying for a high-risk patient with breast cancer within the next five years was 2.69 to 3.04 times of that for a low-risk patient. This is the point that traditional survival models are not able to provide any information about it. We provided predicted five-year (window=5) dynamic probabilities for dying outcome based on the landmark model for the breast cancer patients which was smaller than those of the KM predictions for all time points indicating that there is predictive information in the prognostic index calculated by covariates shown in Table 1. In the present study, ER and PR were among the variables used in calculating prognostic index. While some studies have indicated that ER and PR do not show significant effects on survival of the patients with breast cancer (22–24), several studies have shown that ER and PR do affect the survival of these patients significantly (12, 25, 26). Moreover, HER2 was another important variable included in prognostic factor that was indicated by other studies to have an impact on the survival of breast cancer patients (27, 28). For a complete discussion about the role of these hormones in breast cancer, readers are referred to (2). Other variables included in prognostic index were stage, grade, metastasis, pathological type and surgical approach. All these variables have been reported to be significant in several studies and not to be in some others (25, 29, 30). The informative performance of the prognostic index was evaluated using AUC(t) (time-dependent area under ROC) and Brier Score. It was indicated that including this index in prediction of survival of the patients improved the prediction power of the model dramatically compared with the crude model over time. Generally, the landmark model showed promising performance in predicting survival or probability of dying for breast cancer patients in this study in a predefined window. Therefore, this model can be used in other studies as a useful model for investigating the survival of breast cancer patients. By using this technique physicians are able to give patients information about the probability of surviving in a sliding window of time (18). Nevertheless, the performance of the used model should be evaluated in other diseases.

There were some limitations in the present study. First, information of the patients used was based on a registry-based retrospective study that can introduce potential biases in the estimation of parameters. Second, the existence of censoring in the data may lead to overestimation (underestimation) of the obtained results. Third, due to unknown pathology, the number of 119 breast cancer cases was excluded. Fourth, incomplete data was another important issue for metastatic status. Despite these limitations, as this study was conducted in a middle-income country, the results can provide a dynamic point of view in prediction of the prognostic effect of immunohisto-chemistry markers on breast cancer.

## Conclusion

Generally, the landmark model showed promising performance in predicting survival or probability of dying for breast cancer patients in this study in a predefined window. Therefore, this model can be used as a useful model for investigating the survival of breast cancer patients.

## Ethical considerations

Ethical issues (Including plagiarism, informed consent, misconduct, data fabrication and/or falsification, double publication and/or submission, redundancy, etc.) have been completely observed by the authors. This study was approved by the Research Ethics Committee of Hamadan University (No. IR.UMSHA.REC.1398.568).
